# Characterization and Comparative Investigation of Hydroxyapatite/Carboxymethyl Cellulose (CaHA/CMC) Matrix for Soft Tissue Augmentation in a Rat Model

**DOI:** 10.1021/acsomega.4c01503

**Published:** 2024-07-12

**Authors:** Erkan Karatas, Kubra Koc, Mehmet Yilmaz, Halil Murat Aydin

**Affiliations:** † Department of Molecular Biology and Genetics, Erzurum Technical University, 25100 Erzurum, Turkey; ‡ Department of Biology, Faculty of Science, Ataturk University, 25240 Erzurum, Turkey; § Department of Chemical Engineering, Ataturk University, 25240 Erzurum, Turkey; ∥ Bioengineering Division, Institute of Science, Hacettepe University, 06800 Ankara, Turkey; ⊥ Centre for Bioengineering, Hacettepe University, 06800 Ankara, Turkey

## Abstract

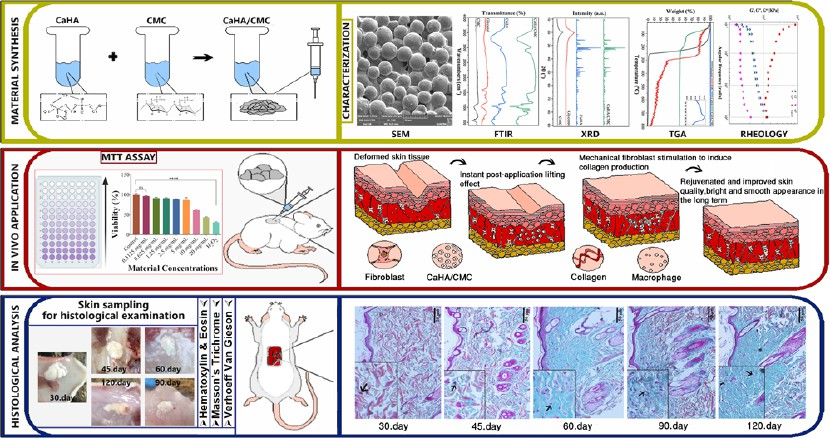

This study endeavors to develop an injectable subdermal implant material tailored for soft tissue repair and enhancement. The material consists of a ceramic phase of calcium hydroxyapatite (CaHA), which is biocompatible, 20–60 μm in size, known for its biocompatibility and minimal likelihood of causing foreign body reactions, antigenicity, and minimal inflammatory response, dispersed in a carrier phase composed of carboxymethyl cellulose (CMC), glycerol, and water for injection. The gel formulation underwent comprehensive characterization *via* various analytical techniques. X-ray diffraction (XRD) was employed to identify crystalline phases and investigate the structural properties of ceramic particles, while thermogravimetric analysis (TGA) was conducted to evaluate the thermal stability and decomposition behavior of the final formulation. Scanning electron microscopy (SEM) was utilized to examine the surface morphology and particle size distribution, confirming the homogeneous dispersion of spherical CaHA particles within the matrix. SEM analysis revealed particle sizes ranging from approximately 20–60 μm. Elemental analysis confirmed a stoichiometric Ca/P ratio of 1.65 in the hydroxyapatite (HA) structure. Heavy metal content exhibited suitability for surgical implant use without posing toxicity risks. Rheological analysis revealed a storage modulus of 58.6 and 68.9 kPa and a loss modulus of 21.7 and 24.8 kPa at the frequencies of 2 and 5 Hz, respectively. 150 μL of sterilized CaHA/CMC was injected subcutaneously into rats and compared with a similar product, Crystalys, to assess its effects on soft tissues. Skin tissue samples of rats were collected at specific intervals throughout the study (30, 45, 60, 90 and 120 days), and examined histologically. Results demonstrated that CaHA/CMC gel led to a significant increase in dermal thickness, elastic fibers, and collagen density. Based on the findings, the formulated CaHA/CMC gel was found to be biocompatible, biodegradable, nonimmunogenic, nontoxic, safe, and effective, and represents a promising option for soft tissue repair and augmentation.

## 1 Introduction

Clinical manifestations such as tissue loss, subcutaneous tissue atrophy, and connective tissue weakness may gradually manifest in soft tissues due to various skin conditions, prolonged sun exposure, trauma, nutritional deficiencies, or natural aging processes. The progressive aging phenomenon entails a gradual degradation of the skin’s extracellular matrix (ECM) and a decline in both the quantity and functionality of fibroblasts, resulting in the clinical presentation of progressive wrinkles, adipose tissue loss, and skin texture degradation.^1^ The ECM acts as an acellular scaffold, providing structural integrity of tissues.^2^ Despite lacking cells, a robust ECM is intricately connected to a diverse network of cells, collectively facilitating tissue homeostasis. The majority of the ECM is composed of key elements such as laminin, glycosaminoglycans, proteoglycans, collagen (types I, II, III, IV), and elastin.^3^ Collagen I and collagen III within the skin’s ECM chiefly confer structural stability, serving as the primary architectural framework for the cellular organization. Elastin, another integral component of ECM, contributes significantly to skin’s elasticity, resilience, and pliability.^4^ Moreover, proteoglycans and hyaluronic acid (HAc) play essential roles in maintaining the skin's viscoelastic properties, ensuring its smoothness, and optimizing its hydration levels.^5^

Fibroblasts and keratinocytes stand as the dominant cell types within the skin’s ECM. However, with age, factors such as matrix metalloproteinases (MMPs) and environmental stressors such as ultraviolet radiation, smoking, and chemical exposure elevate MMP levels along with reactive oxygen species, leading to gradual degradation of the skin’s ECM. Over time this degradation leads to a decline in the skin’s structural integrity and functionality.^5,6^ The primary factor driving cellular aging is the degradation of collagen. When the rate of collagen degradation surpasses its renewal rate, aging ensues.^7^ Moreover, congenital skin and connective tissue disorders may manifest similar clinical presentations.^8^ To address these clinical manifestations, systemic administration of nutritional support products is recommended to rectify defects, diminish skin wrinkling, restore fullness or turgor/tonus loss, and regenerate soft tissues in cases of atrophy.^9^ Regenerative procedures are increasingly incorporating biologically derived treatments such as growth factors, allogeneic adipose matrices, exosomes, stem cells, fibrin, and platelet-rich plasma.^10,11^ Furthermore, numerous materials have been developed for external application to the skin or as dermal fillers. Despite the availability of commercial products, substantial efforts persist in developing novel and more effective materials.^12^

The field of materials science has been researching potential augmentation materials for various organs for over a century. Initial attempts with paraffin yielded suboptimal outcomes.^13^ However, significant progress in soft tissue augmentation were achieved in 1983 with autologous fat transfer. Although materials, such as silicone, were proposed, they were eventually discarded due to the emergence of serious complications.^14^ Researchers at Stanford University reported their initial studies of injectable collagen in the 1970s, considering biodegradation and biocompatibility concerns. Subsequent refinements led to its approval by the US Food and Drug Administration about a decade later.^15^ Following this milestone, research in the field gained substantial momentum, and soft tissue augmentation materials were categorized as either biological or synthetic.^16^ Biological materials consist of bovine collagen, hyaluronic acid, and oils, while synthetic materials include bacterial HAc, poly(methyl methacrylate) (PMMA) microspheres, and hydroxyapatite (HA). With the growing popularity of treatments such as Botox and other similar procedures, the field of injectable soft tissue augmentation continues to expand and evolve. Certain injectable fillers containing exogenous materials have shown remarkable potential to stimulate ECM regeneration. These therapies offer a minimally invasive approach to soft tissue augmentation and revitalization, resulting in minimal downtime. As a result, the demand for such procedures has increased significantly in recent years.^16,17^ With the emergence of various new injectable fillers, it is imperative to assess their composition and properties to make an optimal choice aligned with target tissue requirements. Materials for soft tissue augmentation are expected to provide long-term structural integrity, biocompatibility, proliferative, and regenerative stimulation to achieve the desired anatomical quality, non-migratory behavior from the implant site, and a low side effect and complication profile.

The developed material is a gel composed of calcium hydroxyapatite (CaHA) and carboxymethyl cellulose (CMC). CaHA, a bioceramic consisting primarily of calcium and hydroxyapatite, closely mimics the molecular composition of endogenous HA found in bone and dental structures. Over the past two decades, it has been extensively utilized in orthopedics, dentistry, and otolaryngology to rectify bone deformities.^18^ Unlike other fillers, polymers such as polycaprolactone (PCL), poly(methyl methacrylate) (PMMA), and poly-l-lactic acid (PLLA) bioceramics offer numerous advantages. CaHA is fully biodegradable, has exceptional stability, and can last for up to 30 months.^19^ Additionally, the hydroxyapatites that form CaHA have superior thermal properties, with a higher sintering temperature of approximately 1000 °C, outperforming polymers.^20^ Polymeric fillers, in contrast, have glass transition temperatures that lie within or near energy-based device-induced or physiological temperatures. Thermal stability is critical for combination treatments involving energy-based devices as localized heating can cause polymeric fillers to deform, potentially reducing their efficacy or increasing immune cell recruitment.^21^ Long-term tissue regeneration requires careful consideration of the cell and protein adhesion to microsphere surfaces. Microspheres with hydrophilic surfaces are preferred as they facilitate protein and cell attachment. Conversely, PLLA polymers are relatively hydrophobic, while CaHA microspheres are generally hydrophilic.^22^ The synthetic CaHA used in this study consists of uniform microspheres with diameters ranging from 20–60 μm produced by a precipitation and sintering process. Furthermore, several studies report CaHA ceramics as biomaterials that do not exhibit antigenicity, do not induce foreign body reactions, and are highly biocompatible.^23,24^

Cellulose-based materials are commonly used in biological applications due to their superior properties, such as high-order self-assembly, lower antigenicity content, and enhanced compatibility with established technologies. CMC, a cellulose derivative synthesized by chemically modifying cellulose’s noncrystalline regions with alkylating reagents,^25^ is a water-soluble, biodegradable, and non-toxic material^26−29^ that has exceptional film-forming capabilities.^30,31^ It offers unique properties for emulsification, thermal film formation, binding, gelling, coating, suspending, and thickening.^32−35^ Its polyelectrolyte nature makes it responsive to changes in the ionic strength and pH, enhancing its compatibility when combined with various polymeric substances. This property is particularly important in the preparation of hydrogels, nanoparticles, and biomaterial scaffolds for drug encapsulation.^36,37^ CMC is a commonly utilized material in the fields of biotechnology, tissue engineering, and pharmaceuticals owing to its biodegradability and biocompatibility.^33,35^ In regenerative medicine, CMC is extensively utilized because of its organic origin, outstanding biocompatibility, low potential to trigger inflammatory reactions, and ability to promote cell growth.^28,38^ Furthermore, CMC is biodegradable and naturally eliminated from the body within 6 to 8 weeks.^30^ CMC stands out as a dermal filler gel because of its shear-thinning fluid behavior.^38,39^

This study introduces an innovative injectable gel for soft tissue repair and augmentation comprising spherical HA particles suspended in the CMC. This formulation aims to facilitate tissue repair by stimulating the formation of new skeletal elements such as collagen and elastin, and methylcellulose can be absorbed by the body and replaced by collagen within 2–3 months. In the final stage of the gradual particle disintegration, complete elimination can be achieved by phagocytosis, as demonstrated by numerous researchers.^40−42^ This exceptional material was prepared with a proprietary formulation and was analyzed for its morphological, chemical, thermal, and rheological properties. Our final formulation was tested *in vitro* and subcutaneously on a rat’s dorsum and compared with a commercial filler containing CaHA. The filler response was assessed under *in vivo* conditions from histological and histomorphometric perspectives.

## 2 Experimental Section

### 2.1 Materials

CaHA (BMT Group, Turkey), CMC (Sigma-Aldrich, Darmstadt, Germany), and glycerol (Sigma-Aldrich, Darmstadt, Germany) containing the components of the synthesized biomaterial were provided. In addition, the commercial product Crystalys (Lod, Israel), which is currently used clinically for soft tissue augmentation, was obtained for comparative evaluation of the *in vivo* performance of the biomaterial. Hematoxylin and eosin (Merck, Rahway, NJ), Verhoeff-Van Gieson (Sigma-Aldrich, Darmstadt, Germany), and Masson’s trichrome (Scytek, Logan, UT) stains and Entellan and xylene (Merck, Rahway, NJ) were used as received. HDF (Human dermal fibroblast) (Cat. No. M2267, Cell Biologics) cell lines were obtained from the Eastern Anatolia High Technology Application and Research Center (DAYTAM) (Ataturk University, Erzurum, Turkey).

### 2.2 Preparation of CaHA/CMC Augmentation Gels

The material was prepared in two stages. In the first stage, a mixture consisting of 30% glycerol, 70% deionized water, and 2.0% sodium carboxymethyl cellulose (NaCMC) (calculated in proportion to the combined weight of glycerol and water) was prepared as the carrier phase of the augmentation material, and this mixture was slowly added to deionized water mixed on a magnetic stirrer and allowed to mix at medium speed for 30 min. In the second stage, the glycerol/NaCMC gel prepared in the first stage and spherical, smooth CaHA particles were mixed 60 to 40%, w/w, at low speed to form a homogeneous suspension. Finally, the preparations were filled in syringes and sterilized at 121 °C for 21 min.

### 2.3 Characterization of CaHA/CMC Augmentation Gels

X-ray diffraction (XRD), thermal gravimetric analysis (TGA), scanning electron microscopy (SEM), and inductively coupled plasma–mass spectrometry (ICP–MS) were conducted to determine the thermal, chemical, and morphological properties of the CaHA/CMC gel. The material and its components were subjected to XRD analysis to determine the crystalline and amorphous structures. The product and its components were scanned in the range 2θ = 10–90° at a speed of 2°/min using Cu Kα radiation on the PANalytical Empyrean XRD device to obtain XRD patterns. TGA analyses (DTG60/60, Shimadzu) were performed to investigate the thermal stability of the CaHA/CMC gel formulation and its constituents. Heating was conducted in a nitrogen environment at a flow rate of 1 mL/min, applying temperatures ranging from 30 to 800 °C at a rate of 10 °C/min. For all thermal analyses, the equipment automatically computed the percentage of mass lost due to increasing the temperature in a nitrogen environment. The specimens were analyzed by using a Quanta FEG 250 SEM apparatus to ascertain their surface morphology, particle size, and elemental composition. The particle size distribution of CaHA microspheres, counted based on their sizes with percentage ratios in a given area, was analyzed using ImageJ (1.52e, Solvusoft, Chicago, IL) software. Moreover, the ICP–MS method was employed to investigate the probable occurrence of heavy metals in the final gel formulation ranging from 5 to 270 amu in ng/L.

### 2.4 Rheological Properties of CaHA/CMC Augmentation Gels

The fluid characteristics of the gel formulation were evaluated using the TA Instruments ARES Rheometer. The samples subjected to sinusoidal oscillations at 37 °C in the range of 0.1–100 rad/s between two parallel plates with a 40 mm diameter. The phase angle (tan δ) between stress and strain, storage (elastic) modulus, loss (viscous) modulus, complex modulus, and complex and dynamic viscosities were calculated based on the strain amplitude measurements in response to applied stress.

### 2.5 MTT Assay and Biocompatibility

The effects of various concentrations of the developed CaHA/CMC gels on the viability of HDF fibroblasts (Cat. No. M2267, Cell Biologics) were quantitatively determined using the MTT [3-(4,5-dimethylthiazol-2-yl)-2,5-diphenyltetrazolium bromide] assay (Invitrogen, Thermo Fisher Scientific). HDF cell lines were cultured in high glucose DMEM and DMEM/F12 medium supplemented with 100 IU/mL penicillin, 100 μg/mL streptomycin, and 10% fetal bovine serum (FBS). The cells were grown in a 5% CO_2_ and 95% humidity incubator at 37 °C. These cells were seeded in 96-well plates at 1 × 10^4^ cells/mL density in 150 μL of growth medium for 24 h and then treated with different concentrations (0 (Control), 0.3125, 0.625, 1.25, 2.5, 5, 10, and 20 mg/mL) of the developed CaHA/CMC gels. The stock solution of the gels was prepared by dissolving in phosphate-buffered saline (PBS), sonicated for 5 min, and then diluted for application. After 48 h of incubation, 20 μL of 5 mg/mL MTT in PBS was added to each well, and the cells were further incubated for 4 h. The medium and MTT solution were then removed from each well, and 150 μL of a dimethyl sulfoxide (DMSO) solution was added to dissolve the purple formazan crystal product. Samples were removed from the cell cultures, and the absorbance of formazan produced from MTT was detected at λ570 nm by using an ELISA Microplate Reader (Epoch, Biotech). Cell viability was calculated using the following formula, assuming the absorbance of the control group as 100%
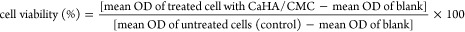


### 2.6 Animals and Experimental Design

The *in vivo* study was conducted at Ataturk University Medical Experimental Application and Research Center (ATADEM), where male Sprague–Dawley rats (weighing 200–230 g) were housed under standard conditions of a 12 h light/dark cycle at 20–22 °C room temperature and 40–50% relative humidity prior to the commencement of the experiment. The rats were fed ad libitum solid chow and water before and during the experimental period. The rats were then randomly divided into three groups: Control, Crystalys, and CaHA/CMC. A total of 90 rats were used in the study, with 30 rats in each of the three groups. Each group was further divided into five subgroups, with 6 rats in each group. These groups were evaluated at distinct time periods of 30, 45, 60, 90, and 120 days. The number of animal groups (*n* = 6) used in the study was calculated using the *t*-test and the G-power software with an 80% power expectation and predicted impact range between groups. Before the procedure, the dorsal area to be injected was shaved.

- The control group was treated with only 0.5 mL of physiological saline subcutaneously, ensuring a comprehensive and objective baseline for the experiment.
- A total of 150 μL of Crystalys filler was subcutaneously administered with a 27-gauge half-inch needle to the dorsal regions of rats in the Crystalys group. The commercial reference, Crystalys, consists of synthetic calcium hydroxyapatite microspheres formulated to a concentration of 55.7% (w/w), suspended in an aqueous carrier gel. Its composition includes calcium hydroxyapatite microspheres of 25–45 μm in diameter (55.7%), glycerin, sodium carboxymethyl cellulose, and phosphate buffer, according to information obtained from the product catalog.
- In the CaHA/CMC group, 150 μL of the newly developed injectable filler was subcutaneously injected into the dorsal regions of rats using a 27-gauge half-inch needle.
- On days 30, 45, 60, 90, and 120 of the study, six rats from each group were randomly selected and sacrificed under sevoflurane inhalation anesthesia to explant the skin and subcutaneous areas where the fillers were applied. The samples were then fixed in a 10% neutral formaldehyde solution.

### 2.7 Histological Assessments

Fixed tissue samples were dehydrated and embedded in paraffin before all staining procedures. Skin samples were then sectioned at 5 μm thickness, deparaffinized in xylene, and stained with hematoxylin and eosin (H&E) (to confirm inflammation and foreign body reaction), Verhoeff-Van Gieson (VVG) (to confirm elastin biosynthesis), and Gomori’s one-step Trichrome (Trichrome) (to confirm collagen biosynthesis), as specified in the manufacturer’s instructions. The thickness of both the epidermis and dermis was measured by analyzing slides stained with H&E. In H&E staining, cell nuclei are stained purple-blue, while the cytoplasm is stained pink. Elastic fibers, one of the parameters evaluated in this study, were examined by VVG staining. VVG elastin staining detects elastic fibers in tissues by staining cell nuclei and elastin fibers black, collagen red/orange, and other tissue elements yellow. A trichrome staining protocol was utilized to differentiate collagen components in tissues. The trichrome staining protocol stains the cytoplasm, keratin, and muscle fibers red, collagen green, and cell nuclei purple-blue or black. Five random microscopic fields were selected for analysis. Collagen density, fibroblast density (average cell number), epidermis, and dermis thickness were measured in the skin tissue sections to conduct in-group and intergroup evaluations. Quantitative evaluations of the collagen fiber and dermis-epidermis thickness measurements in all of the groups were performed using ImageJ software. Measurements were made in relation to previous studies.^43,44^ Elastic fibers were examined under a trinocular microscope (Zeiss, Axio5, Germany) by counting all of the fibers present on the surface of each section and quantifying them at ×40 magnification. The mean value of all tissue samples was then calculated to give the percentage of fiber density per square micrometer. Quantitative evaluations of elastic fiber patterns in all groups were also performed using ImageJ software.^45^

### 2.8 Statistical Analysis

Statistical analyses of histomorphometric measurements and the MTT assay were conducted using GraphPad Prism 9.5.1 software (GraphPad, La Jolla, CA). The normality of the data was assessed using the Kolmogorov–Smirnov test. Descriptive statistical analyses were performed, reporting means ± the standard deviation. Two-way analysis of variance (ANOVA) and post-hoc Tukey tests were applied for intragroup and intergroup comparisons. *P*-values less than 0.05 with a 95% confidence interval were considered statistically significant.

## 3 Results

### 3.1 XRD Findings

**Figure 1:**
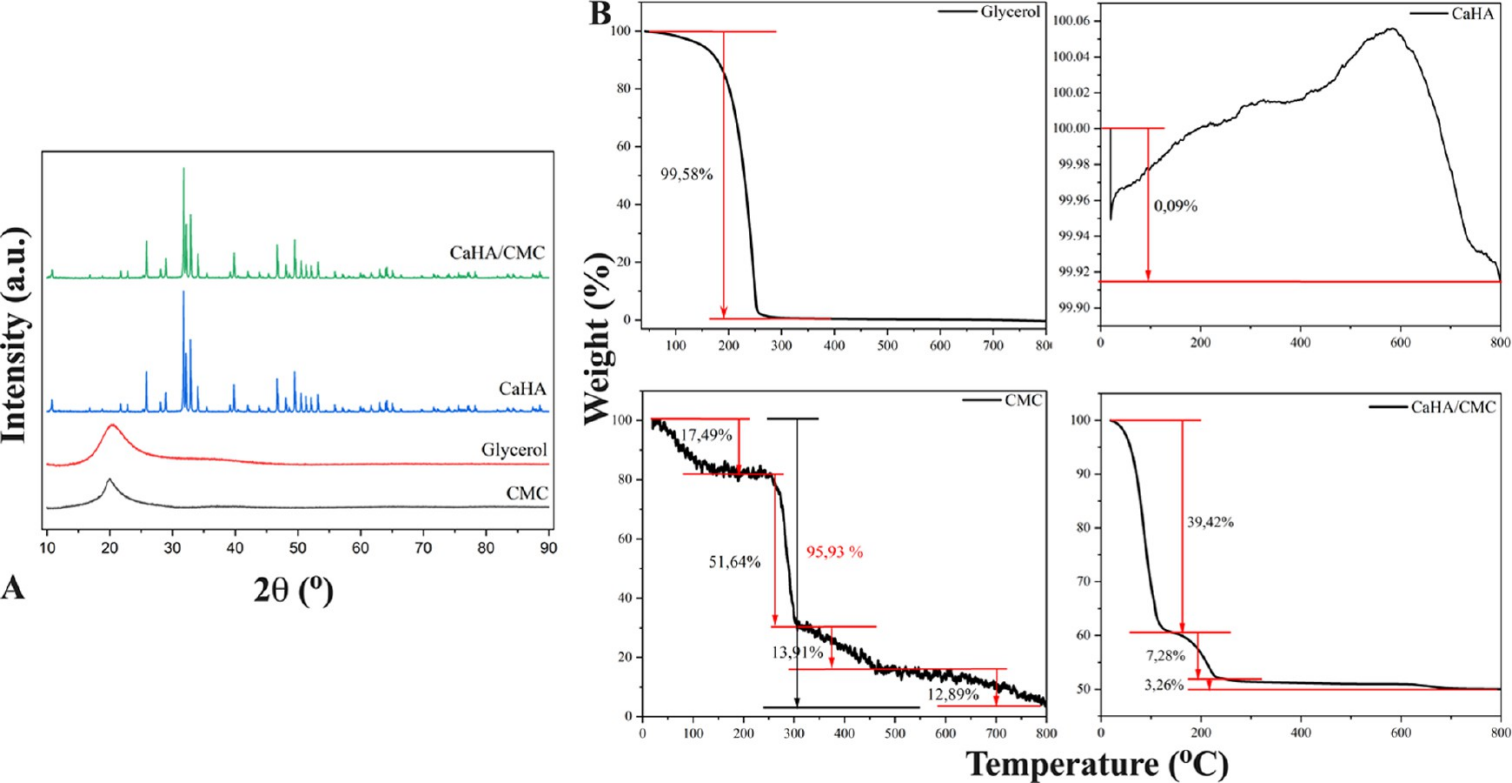
XRD patterns of the material (CaHA/CMC) and its components (CaHA, CMC, and glycerol) (A), and TGA thermogram of the material and its components (B).

Figure 1A displays the XRD patterns of CaHA/CMC and its constituents: CaHA, CMC, and glycerol. The diffractograms exhibit sharp peaks at 26, 29, 32, 34, 40, 47, and 50–55°, indicating the robust crystal structure of HA.^46^ High intensity peaks indicate highly crystalline regions. The XRD pattern of the CaHA/CMC composite loaded with HA confirms a pure apatite phase and clearly shows the peak characteristics of pure HA, which corresponds to the reference (JCPDS) pattern no. 09–432. The equivalence of peak intensity observed in both the CaHA/CMC phase and the CaHA phase suggests that the constituents of the final product do not interact with the crystal structure of CaHA. The lack of interaction confirms that the physicochemical properties, including the HA morphology, functional groups, and surface charge, remain unaffected. Therefore, the bioactive phase of the gel material, composed of hydroxyapatite molecules, would be functionally effective in the tissue.

The broad, nonsharp peaks observed at 2θ = 20° suggest that the other components, glycerol and CMC, lack a crystal structure or have a low crystal structure content. Thus, it can be inferred that the carrier polymer phase components have amorphous structures and possess the capability to effectively serve as carriers in rheological terms. This outcome agrees with the literature’s findings.^30,47^

### 3.2 TGA Findings

The results of thermogravimetric analysis of the synthesized material and its components are shown in Figure 1B. The glycerol employed in the synthesis phase underwent a single-step decomposition, with most of the mass loss occurring at around 280 °C. Further loss of the remaining negligible mass occured up to 800 °C. Moreover, CMC, one of the carrier phase components, had a mass loss in three stages. During the experiment’s first phase, CMC experienced a mass loss of 17.49% between 50 and 120 °C.^48^ In the subsequent and main decomposition stage, there was a loss of 51.64% between 250 and 300 °C, with the highest decomposition temperature peaking at 279 °C.^49^ The loss of 26.8% mass in the final stage is attributed to the evaporation of structural water after the degradation of the main structure.^50^ CaHA, another substance examined in the study, did not decompose at temperatures up to 800 °C.^51^

The synthesized CaHA/CMC filler underwent mass loss in three stages. The first stage resulted in a mass loss of 39.42%. The mass loss between temperatures of 50 and 150 °C was due to the evaporation of moisture within the material sample and some glycerol decomposition. During the third stage, the structure experienced a mass loss of 3.26%, while CO_2_ removal peaked at temperatures ranging from 230 to 700 °C. The mass loss continued until it reached the maximum decomposition temperature of 279 °C. The presence of carboxyl groups in the CMC led to decarboxylation within a specific temperature range during this stage. A certain degree of mass loss was attributed to the evaporation of water from the basic structure.

### 3.3 SEM Findings

**Figure 2:**
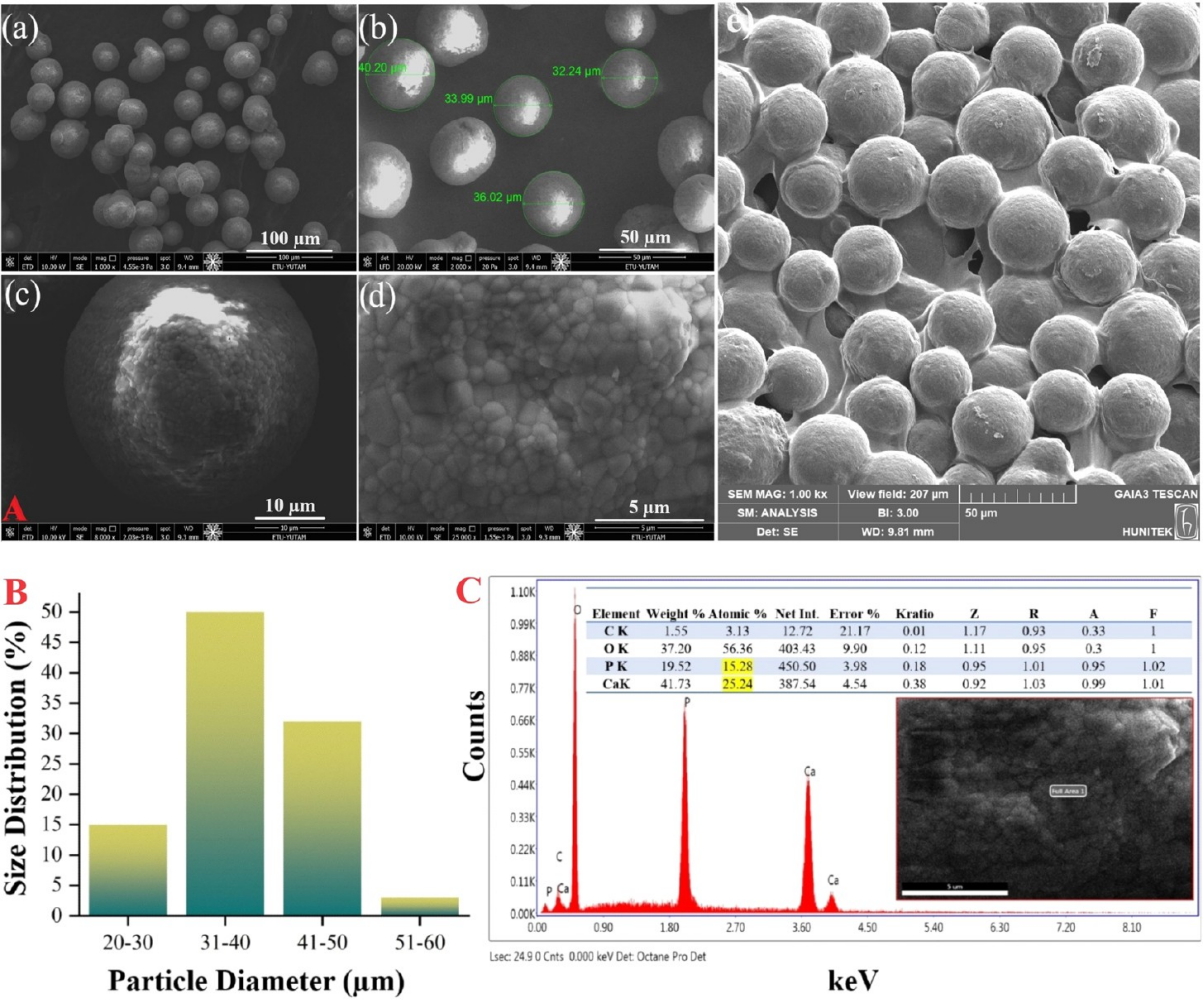
CaHA microspheres (A), SEM image, 1000× (a), SEM image of size distribution, 2000× (b), SEM image of a particle, 8000× (c), SEM image of particle’s surface in detail, 25,000× (d), SEM image of CaHA/CMC gel (e). Particle size distribution of CaHA microspheres (B). EDS analysis results of CaHA, inset: the stoichiometric ratio of Ca/P (C).

Figure 2A shows SEM images of HA microspheres and a freeze-dried CaHA/CMC gel, revealing spherical HA particles with a homogeneous distribution. The majority of particles were 30–50 μm in diameter, comprising over 80% of the sample’s particle sizes shown in Figure 2B. Furthermore, the HA particle surfaces had pores and cavities between 2 and 5 μm, with optimal properties reported in the literature for implant tissue integration.^52,53^ Studies using polycaprolactone (PCL) microspheres as dermal fillers have emphasized the suitability of microsphere-structured materials for dermal filling in terms of size, surface properties, and inability to be phagocytosed.^54,55^ In addition, related literature states that the porosity and cavities associated with the HA used in the developed gel formulation enhance its interaction with the microenvironment.^56^

During analysis, using the SEM device’s elemental analysis equipment, one of the randomly selected HA microspheres was found to have a stoichiometric Ca/P ratio of 1.65 on its surface, as shown in the inset of Figure 2C. It has been reported in many studies that HA, with a ratio of 1.67 in the literature, has a hexagonal structure and the chemical formula Ca_10_(PO_4_)_6_(OH)_2_, is bioactive and biocompatible and is commonly used in biomedical applications.^57,58^ The HA utilized in this study was in close stoichiometric ratios, as shown in the inset of Figure 2C, indicating the contribution of the ceramic phase to the efficacy, biocompatibility, and bioactivity of our injectable final formulation.

### 3.4 ICP–MS Findings

The developed soft tissue augmentation material was examined by an ICP-MS assay, and the levels of analyzed elements were found to be below the detection limits of the instrument. When compared to the maximum acceptable trace levels from the ASTM F1185–03 standard elements, the levels for arsenic, cadmium, mercury, and lead were 0.033, 0.002, 0.004, and 0.00033 ppm, respectively (Table 1). Based on these values, it is evident that the heavy metal content of the developed material is below the standard limits and does not carry any risk of toxic effects. This result suggests that the formulated material is appropriate for use as a surgical implant and does not pose any risks to the organism, even with prolonged use.

**Table 1:** Trace Element Concentrations of Heavy Metals in the Formulated CaHA/CMC Gel^a^

element (ppm)	As	Hg	Cd	Pb
ASTM F1185–03 standard specification for composition of hydroxylapatite for surgical implants	<3	<5	<5	<30
CaHA/CMC gel formulation	<0.1	<0.02	<0.01	<0.01

a As: Arsenic, Hg: Mercury, Cd: Cadmium, and Pb: Lead.

### 3.5 Rheological Properties of CaHA/CMC Gels

The rheological properties of CaHA/CMC were evaluated to determine the material’s ability to integrate with surrounding soft tissue and alter the volume of the injected anatomical layer. The properties assessed included phase angle (δ); storage (*G*′), loss (*G*″), and complex (G*) moduli; and complex and dynamic viscosities.

**Figure 3:**
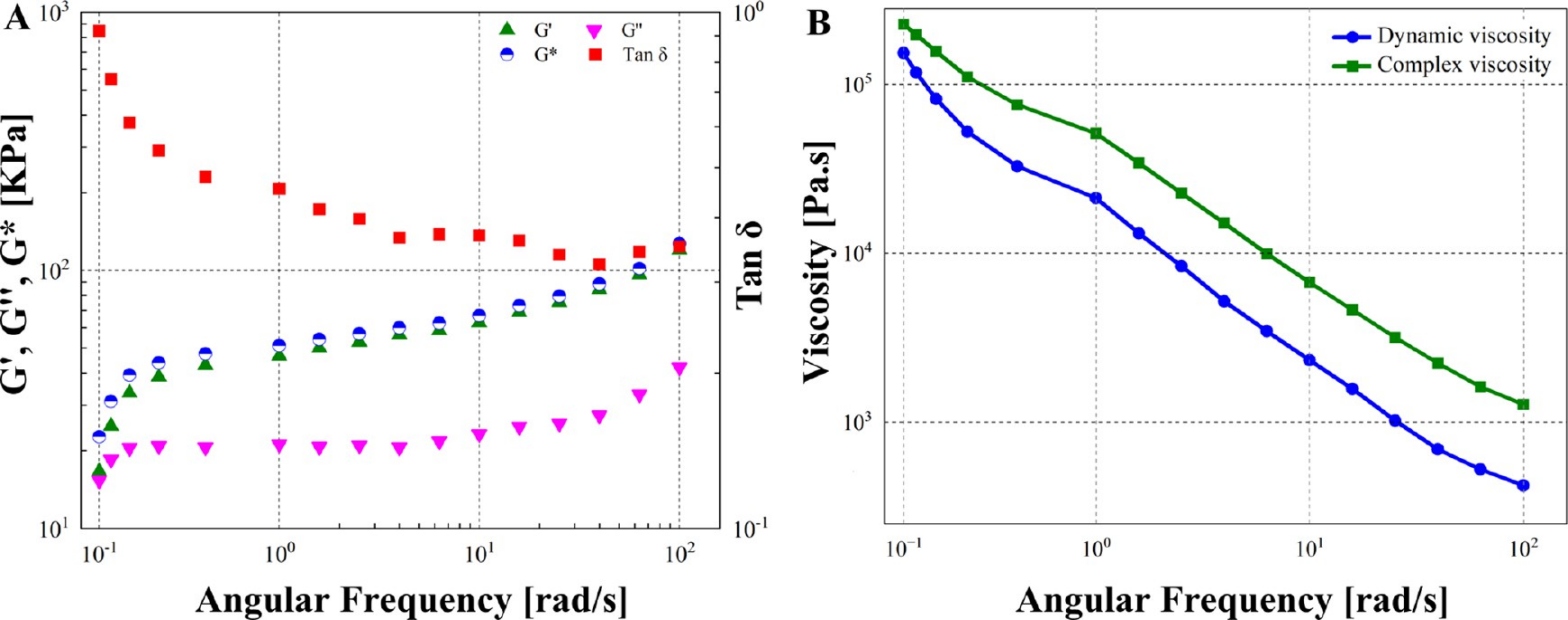
Elastic, viscous, and complex moduli and tan δ values of CaHA/CMC gel (A), and complex and dynamic viscosities of CaHA/CMC gel (B).

Figure 3A demonstrates that as the angular frequency increases, the elastic component (*G*′) of the material contributes more to the complex modulus (G*), indicating that the CaHA/CMC gel can resist deformation when exposed to external forces within its anatomical placement. As frequency increases, the storage modulus corresponds with the complex modulus, suggesting that the material displays viscoelastic solid behavior. Likewise, at low frequencies, the viscous component (*G*″) contributes more to the complex modulus of the material than at relatively high frequencies.

The loss tangent, derived from both the elastic (*G*′) and viscous (*G*″) moduli, is a crucial parameter for analyzing the rheological properties of gels, hydrogels, or materials used for soft tissue augmentation.^59,60^ The ratio of these moduli (*G*″/*G*′) determines the loss tangent (tangent delta), which informs the material’s behavior under deformation. The ratio of viscosity to elasticity, known as tan δ, indicates the elasticity of a material. If an injectable has a tan δ < 1, it functions as a material with high elasticity, but if tan δ > 1, it is similar to a viscous liquid. Clinically, a lower tan δ is related to a high *G*′.^61^

Figure 3A depicts that the developed CaHA/CMC gel material demonstrates a higher *G*′ value than *G*″ at all tested frequencies, indicating a loss factor of less than 1 at all measured frequencies. This outcome proves that the developed CaHA/CMC gel primarily exhibits elastic behavior. Additionally, lower values of tan δ signify that the filler material is more solid or gel-like. This parameter determines the elasticity of the CaHA/CMC filler and whether it can be injected superficially or more deeply. A filler with a tan δ value close to 1 has a low elastic and high viscous component. The tan δ value of the developed CaHA/CMC gel in the frequency range of 0.1–100 rad/s was less than 1, suggesting that the gel formulation exhibited gel-like elastic behavior at low forces and viscous behavior at high forces.

Figure 3B shows the decrease in complex and dynamic viscosities of the CaHA/CMC gel with an increase in frequency, suggesting that the gel can be easily applied to soft tissues with minimal force. This feature enables gentle application to soft tissues, improving patient comfort and clinical effectiveness.

### 3.6 Cytotoxic Effect of CaHA/CMC Gel on HDF Cells

**Figure 4:**
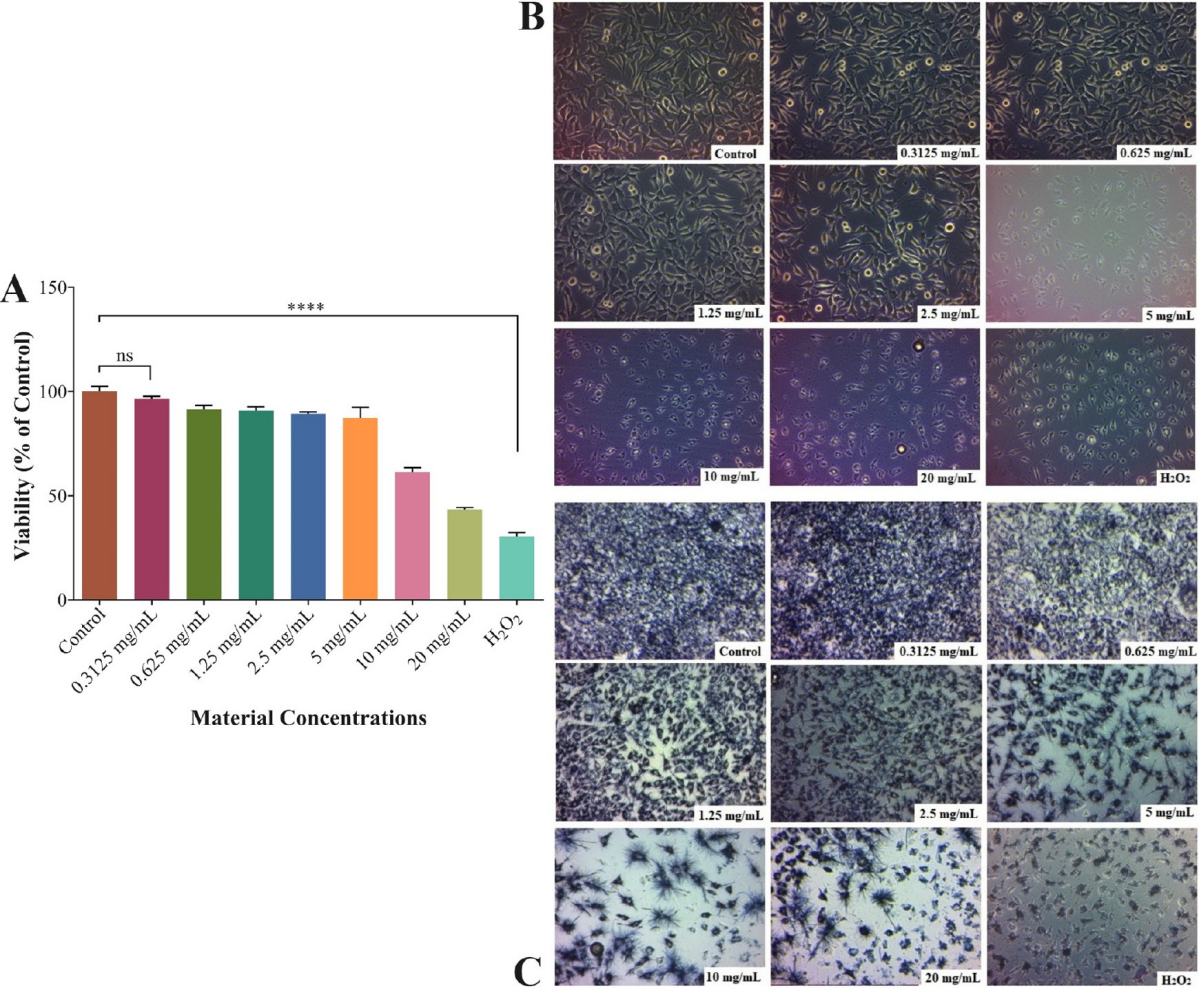
Cell viability (A), HDF cell morphology treated with CaHA/CMC gels for 48 h (B), and HDF cell morphology treated with CaHA/CMC gels for 48 h after 4 h MTT treatment (C). Results are presented as percentages of cell viability calculated relative to the control group without any substance. Error bars correspond to the standard error of the mean of three replicate experiments. ns: not statistically significant and ****: *p* < 0.0001 indicates significant differences between the control and other groups studied by Tukey’s multiple range tests.

The viability of human dermal fibroblasts (HDF) was measured at 7 concentrations ranging from 0–20 mg/mL of the CaHA/CMC gel. Figure 4A shows that HDF cells treated with CaHA/CMC gels at concentrations between 0 and 10 mg/mL for 48 h did not exhibit a significant cytotoxic effect compared to the control group, suggesting no significant change in cell viability. At 0.3125 mg/mL, cell viability was 96.44%, decreasing to 91.40% at 0.625 mg/mL, 90.89% at 1.25 mg/mL, 89.38% at 2.5 mg/mL, 87.34% at 5 mg/mL, and 61.38% at 10 mg/mL. Cell viability decreased as the material concentration increased. The highest concentration tested, 20 mg/mL, significantly reduced cell viability by 43.42% during the application period. The IC_50_ value for HDF cells was calculated as 18.93 mg/mL. Treatment with H_2_O_2_ (300 μM) for 48 h led to a reduction in cell viability of up to 30.47% in the same cell line.

Figure 4B shows optical microscope images of HDF cells treated with CaHA/CMC in the concentration range of 0–20 mg/mL for 48 h. The results indicate that the cell morphology becomes progressively rounder with increasing CaHA/CMC dose. Furthermore, the adhesion strength of cells decreases with increasing dose and the dense HDF population in clusters becomes sparser. Figure 4C shows microscopic images of HDF cells treated with CaHA/CMC gels at concentrations ranging from 0–20 mg/mL for 48 h and then exposed to MTT solution for 4 h. The images indicate a decrease in the number of formazan crystals formed, suggesting a decrease in mitochondrial activity and HDF cell viability. A decrease in color intensity was observed with increasing CaHA/CMC dose.

### 3.7 Effects of Developed CaHA/CMC Gel on Histological Changes in Rat Skin

#### 3.7.1 Evaluation of Epidermis and Dermis Thickness in Rat Skin

**Figure 5:**
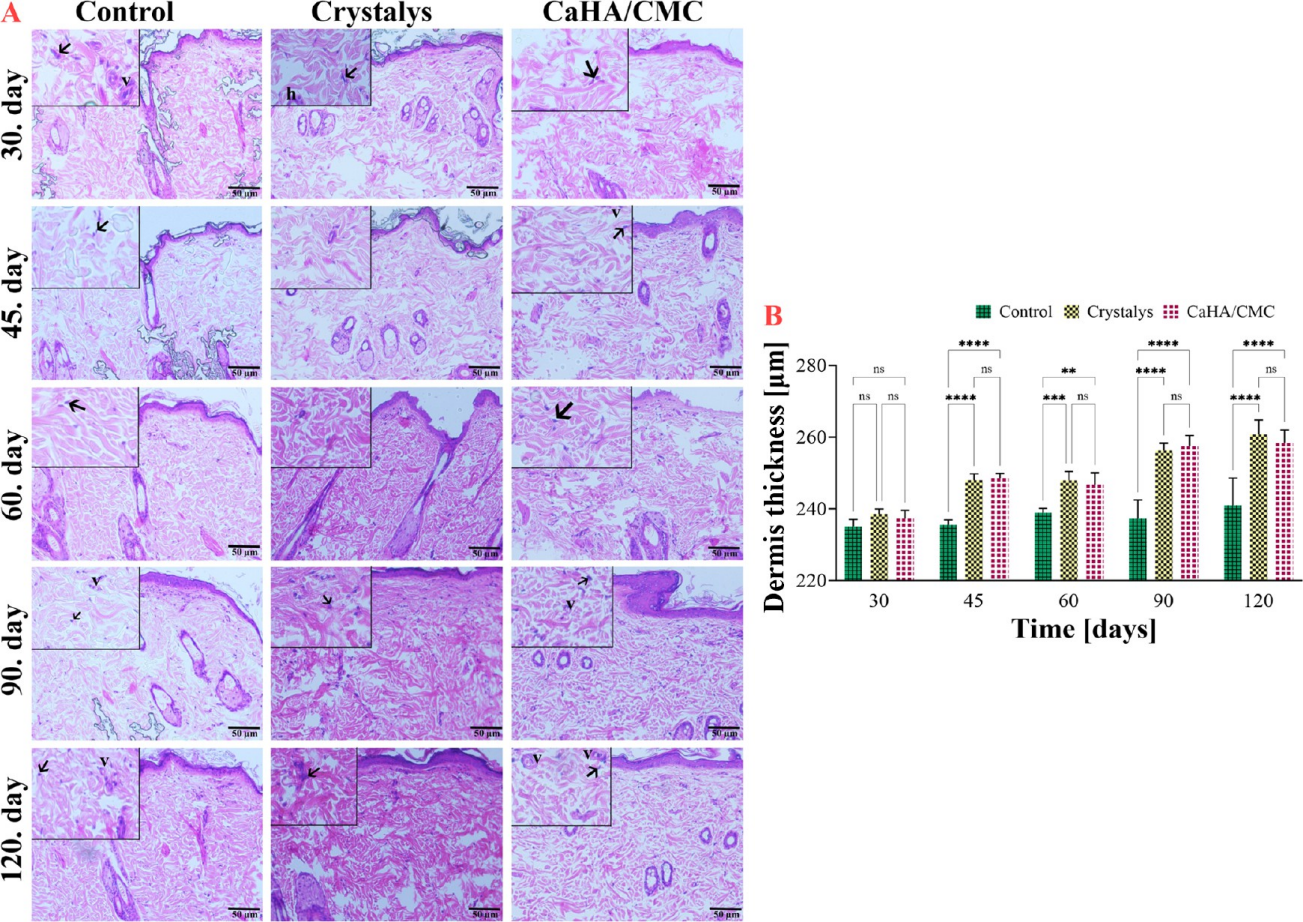
Microscopic images of rat skin samples stained with H&E from the Control, Crystalys, and CaHA/CMC groups on specified time intervals. Fibroblast (arrow), hair follicle (h), blood vessel (v). H&EX20 (A), and mean dermal thickness for specified time intervals (B). Error bars correspond to the standard error of the mean of the dermis thicknesses measured in five separate areas per specimen. ns: not statistically significant, **: *p* < 0.01, ***: *p* < 0.001, and ****: *p* < 0.0001 indicates significant differences between the control and other groups examined by Tukey’s multiple range tests.

Epidermal and dermal thickness were assessed at five different time points using H&E staining, as shown in Figure 5A. Skin thickness was measured from five different regions of the samples, and the results are shown in Figure 5B. No statistically significant differences were observed between the Control, Crystalys, and CaHA/CMC groups when comparing their respective epidermal and dermal thickness measurements on day 30 based on H&E staining results. The data were expected for the intended application in subcutaneous tissue. Mild edema and thickening due to minimal inflammation was expected in all skin layers during the early stages of routine application. It was recognized that this was a pathophysiological process that would subside within a few days, and the epidermis–dermis would return to its natural state.

After 45 days, there were only minor variations in the thickness of epidermis samples from skin tissue between the Control, Crystalys, and CaHA/CMC groups, and no statistically significant differences were found. However, the dermis thicknesses of the Crystalys and CaHA/CMC groups was significantly higher than that of the Control group (*p* < 0.0001). There was no significant difference in the comparison between the applied groups (*p* > 0.05).

In this study, we assessed the thickness of the epidermis and dermis over a 4-month period. The results showed a significant increase in dermal thickness in the application samples compared to the Control group at all time points (*p* < 0.0001). However, no significant increase in epidermal thickness was observed between the groups, including the Control group. The CaHA/CMC group exhibited variations in dermis thickness at different time points, with a gradual increase from day 30 to day 120.

#### 3.7.2 Evaluation of Elastic Fiber Density in Rat Skin Tissue

**Figure 6:**
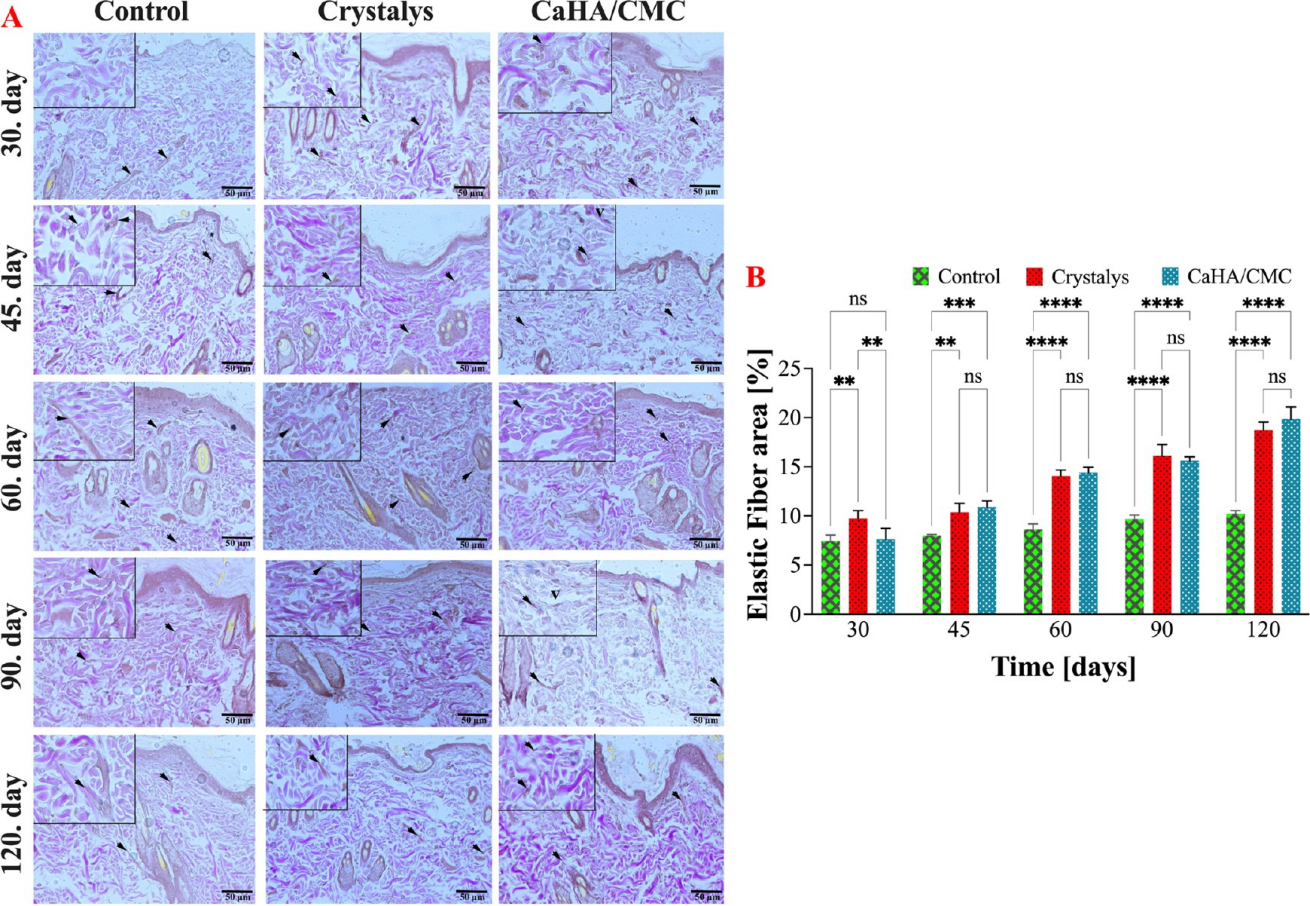
Microscopic images of rat skin samples stained with VVG from the Control, Crystalys, and CaHA/CMC groups on specified time intervals. Rat skin, control group with normal skin histology; Crystalys and CaHA/CMC groups with elastic fiber (arrow). VVGX20 (A), and distribution of mean elastic fiber density on specified time intervals (B). Error bars correspond to the standard error of the mean of the elastic fiber density determined in five separate areas per specimen. ns: not statistically significant, **: *p* < 0.01, ***: *p* < 0.001, and ****: *p* < 0.0001 indicates significant differences between the control and other groups studied by Tukey’s multiple range tests.

Elastic fiber density was analyzed by examining VVG-stained rat skin sections at specific time points in the experimental model, as shown in Figure 6. The results indicate that the Crystalys group had a significantly higher fiber density compared to the Control and CaHA/CMC groups (*p* < 0.01) on day 30. However, there was no significant difference between the CaHA/CMC and Control groups. The study clearly demonstrates that the Crystalys filler had a significantly greater impact on increasing the number of elastic fibers compared to CaHA/CMC. Notably, on day 45, both the Crystalys and CaHA/CMC groups exhibited changes in tissue elastic fiber density compared to the Control group (*p* < 0.001, *p* < 0.0001). The skin tissue samples taken on the 60, 90, and 120 days clearly show that both the Crystalys and CaHA/CMC groups had a significant increase in elastic fiber density compared to the Control group during both periods (*p* < 0.0001). Despite minimal differences between the two groups for the time periods, the results demonstrate the efficacy of both treatments in improving skin elasticity. This outcome supports the effectiveness and success of the newly synthesized CaHA/CMC filler by our proprietary formulation, in activating fibroblasts and biosynthesizing secondary elastic fibers.

Overall, the tissue samples treated with CaHA/CMC gel material exhibited a consistent and significant increase in elastic fiber density compared to the Control group over a 4-month period. The increase was 0.2, 2.93, 5.8, 5.96, and 9.64% at days 30, 45, 60, 90, and 120, respectively.

#### 3.7.3 Evaluation of Collagen and Fibroblast Densities in Rat Skin Tissue

**Figure 7:**
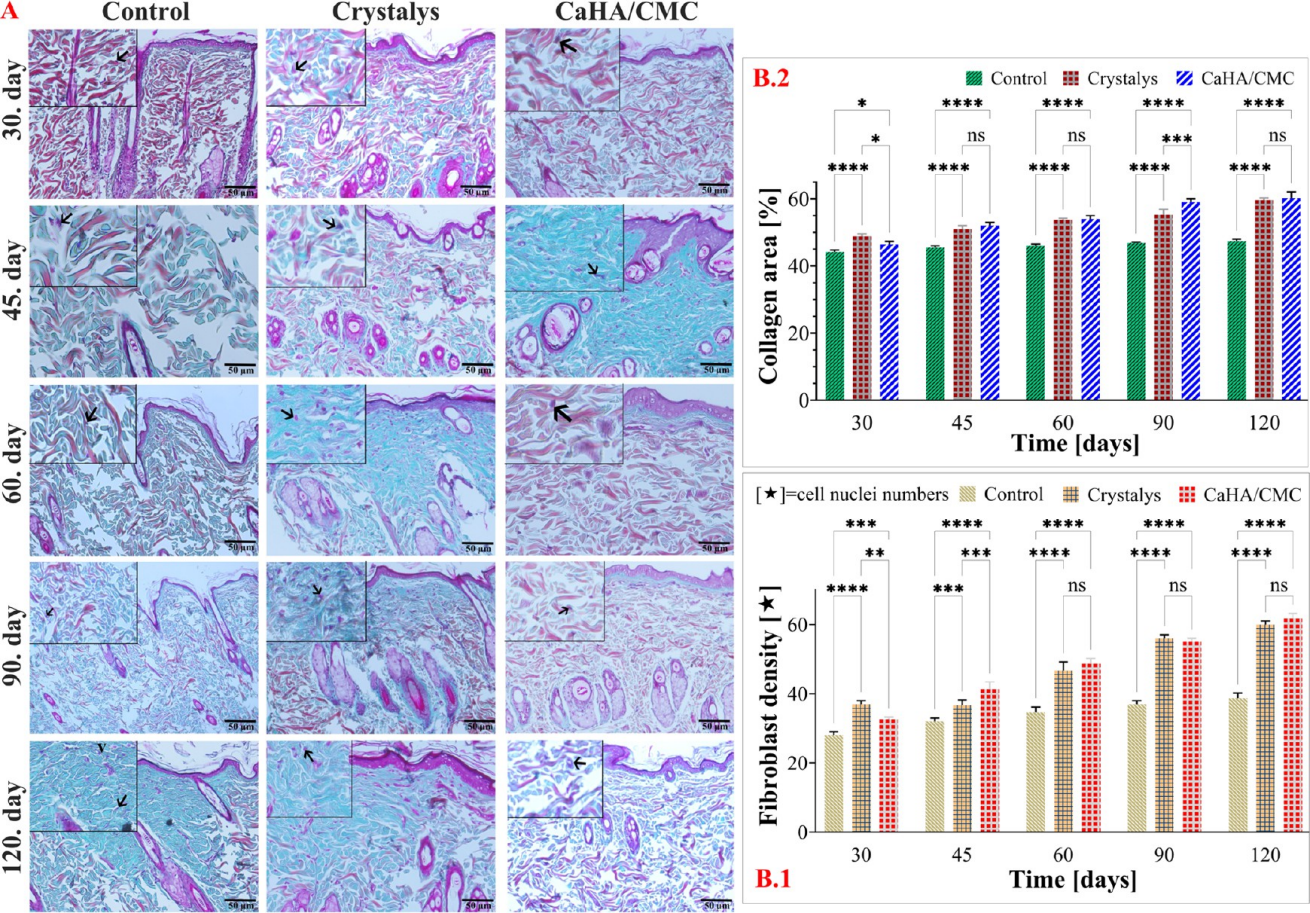
Microscopic images of rat skin samples stained with VVG from the Control, Crystalys, and CaHA/CMC groups on specified time intervals. Rat skin, control group with normal skin histology; Crystalys and CaHA/CMC groups with collagen fiber (asterisk), fibroblast/fibrocyte nucleus (arrow). TrichromeX20 (A), distribution of mean collagen and fibroblast density on specified time intervals (B). Error bars correspond to the standard error of the mean of collagen fiber and fibroblast/fibrocyte nuclei determined in five separate areas per preparation. ns, not statistically significant, *: *p* < 0.05, **: *p* < 0.01, ***: *p* < 0.001, and ****: *p* < 0.0001 indicates significant differences between control and other examined groups by Tukey’s multiple range test.

Evaluation of the microscopic images and statistical analyses presented in Figure 7A,B, indicate that collagen and fibroblast density increased significantly (*p* < 0.0001) in the Crystalys and CaHA/CMC groups compared to the control group in the skin samples collected at 30, 45, 60, and 120 days. It is important to acknowledge that while there may be some differences between the two groups during this time period, they do not appear to be statistically significant. It is worth noting that the samples taken on the 90th day indicated a significant increase in collagen density in the synthesized CaHA/CMC group compared to the Crystalys group. Although an increase in fibroblast density was observed compared to the control group, there did not appear to be a significant difference in fibroblast density between the two groups after 90 days.

Tissue samples treated with CaHA/CMC showed a gradual increase in collagen density over time, with the greatest increase of 12.77% observed on the 120-day. This increase was significantly higher than that in the Control group.

## 4 Discussion

In this study, we characterized our proprietary CaHA/CMC formulation, which incorporates HA microspheres into CMC, a plant-derived polysaccharide commonly used in dermal fillers. The efficacy of our exclusive CaHA/CMC gel formulation for soft tissue repair/augmentation was demonstrated in subcutaneous implantation studies in rats.

Consistent with the literature, our results suggest that CaHA degradation occurs at temperatures above 800 °C.^51^ Our synthesized material maintained pure HA properties without interacting with the CaHA crystal structure. Furthermore, the HA particle surfaces exhibited optimal properties for implant tissue integration, as reported in the literature.^52,53,56^ Studies using polycaprolactone (PCL) microspheres as dermal filler materials have highlighted the advantages of materials with a microsphere structure for dermal filling in terms of phagocytosis resistance, size, and surface properties.^54,55^ In addition, it is known that the pores and cavities of the HA used in our proposed gel material enhance the interaction with the microenvironment.^56^ The HA microspheres were present in the stoichiometric ratios reported in the literature and contributed to the biocompatibility and bioactivity of the final formulation.^57,58^ The HA morphology, functional groups, surface charge, and physicochemical properties of our material were unaffected, and the HA microspheres were found to have optimal properties for tissue integration, suggesting that HA, which is the bioactive phase of our material, could function effectively in human tissues. In addition, the glycerol and CMC in the composition of our product were found to be suitable as a carrier phase, and their application as a gel filler material was physiologically effective. The material was found to be nontoxic in terms of heavy metal content, making it suitable for use as a surgical implant material without posing any risk to the organism during chronic use (Table 1).

The biocompatibility profile of the CaHA/CMC gel on healthy HDF cell lines was assessed through MTT analysis. The findings revealed that only the highest concentration (20 mg/mL) of the applied CaHA/CMC doses reduced the viability of healthy HDF cells by 50%. Based on the findings, it was observed that healthy cells treated with concentrations of up to 10 mg/mL exhibited the most promising viability profile. The MTT analysis indicates that the CaHA/CMC filler is biocompatible, and has no toxic effect on HDF cell viability when applied at optimal doses as determined by the IC_50_ value (Figure 4A). The samples did not exhibit any cytotoxicity at the appropriate doses, indicating their potential suitability for use in soft tissues. It is important to note that these results are consistent with previous research.^62^ Basu et al. reported on the biocompatibility of an electrospun bioengineered soft tissue substitute composed of poly(ethylene oxide) and CMC.^26^ Based on these data and our MTT analysis results, we conclude that our unique CaHA/CMC filler formulation can provide a suitable environment for cell growth and proliferation.

Our specially formulated CaHA/CMC filler demonstrated remarkable success at the injection site, as evidenced by favorable results in collagen and elastic fibers, fibroblast density, and dermis thickness in rat tissue sections. Throughout the study, rats treated with CaHA/CMC gels exhibited favorable anatomical responses without any signs of acute or chronic inflammatory reactions, foreign body reactions, abnormal formations, irregularities, granulation tissue, host tissue necrosis, or other malignant/benign lesions. These observations suggest that Crystalys may have a higher potential to induce collagen production, possibly due to its higher HA content compared to that of our developed material. During our study, collagen and fibroblast densities gradually increased from day 30 to the end of day 120 in tissue samples where CaHA/CMC was applied. This finding underscores the efficacy of the CaHA/CMC gel in promoting fibroblast activation and collagen density over an extended period compared to the positive control. Additionally, evaluation of dermis thickness across different time periods of the CaHA/CMC group revealed consistent gradual increases from day 30 to day 120. This sustained dermal thickening was attributed to the effectiveness of the HA-containing CaHA/CMC filler material. The significant increase in fibroblast, elastic fiber, and collagen density within the dermal tissue likely contributed to indirect dermal thickening and tightening, highlighting the potential of our specially formulated filler material for effective soft tissue augmentation and clinical success. Following subcutaneous filler application, any physiological inflammatory responses observed, such as mild edema and thickening across all skin layers, subsided within a few days, and the epidermis–dermis returned to its normal state. These findings indicate the transient nature of the inflammatory response to early-stage local physical stimulation and further support the safety and biocompatibility of the CaHA/CMC filler *in vivo*.

An experimental study reported a significant increase in collagen fibers in the subcutaneous layer of rats sacrificed 7 days after HA application.^63^ Another study conducted in an experimental canine model found that HA induced more intense new collagen formation at 1 month compared to 5 months.^64^ Similarly, another study observed a greater increase in collagen fibers in the dermis and subcutaneous layer of rat skin samples at day 60. Previous studies have shown a significant increase in collagen density at 2 months after CaHA application.^65,66^ Consistent with these findings, our study showed a significant increase in collagen density at day 30 compared to the control group, indicating active new collagen synthesis during the first month after filler application. Similar results were observed in the positive control group, Crystalys. In the study by Yanatma et al., fibroblast density was assessed based on the number of nuclei. Although there was no significant difference between the control, PCL, and CaHA groups at 2 months after application, a significant difference was reported at 4 months.^65^ When comparing samples at two and four months, the CaHA and control groups showed similar characteristics, while PCL significantly affected fibroblast density.^65^ However, in our study, fibroblast density was significantly higher in both the CaHA/CMC filler material and the positive control material at all sampling periods of 30, 45, 60, 90, and 120 days compared to the control group. It has been suggested that the difference between these data may be due to differences in the HA content and microsphere characteristics of the filler materials used in the two studies. It has been mentioned that in composite materials such as HA-HAc used in filler production, the particle size of HA affects the collagen density. As the size decreases, procollagen activity and collagen production increase along with skin collagen density.^67,68^

In a study comparing nanosized HA and microsized HA, both HA groups were found to be more effective in increasing collagen density than pure HAc. Collagen was shown to form a denser layer in these groups. In our study, HA in the size range of 20–60 μm was used and appears to have the potential to be more effective than pure filler materials, which is consistent with the literature. Previous studies have shown that HAc fillers increase fibroblast activity by comparing the characteristics of active and inactive fibroblasts.^63^ On the other hand, another study attributed the proliferative nature in the experimental HA-HAc composite filler model to HA particles.^67,68^ Consistent with this, our study also showed an increase in the fibroblast density in both our developed CaHA/CMC material and the Crystalys groups, which was related to the increase in collagen.

In various experimental models, HA-based fillers have been found to stimulate dermal fibroblasts in adjacent areas of the application site due to the absorption of interstitial fluid in the first few weeks after subcutaneous injection. In this context, the presence of a granulated endoplasmic reticulum in highly stressed fibroblasts, indicating increased protein synthesis, suggests that hydrogel fillers indirectly trigger fibroblast activation and collagen production through their water-attracting effect from neighboring areas.^69^ Additionally, the accumulation of hydrogel material in the application area triggers fibroblasts due to mechanical stress, thereby increasing collagen production.^63^ As the CaHA/CMC filler material produced by our formulation also possesses hydrogel properties, it may increase collagen density by activating fibroblasts. The application of hydrogel filler material can directly affect fibroblasts while also activating them by absorbing intercellular fluid in neighboring areas, creating tension in neighboring fibroblasts, inducing mechanical stress on fibroblasts in the application area, and promoting fibroblast migration to the application area, ultimately leading to increased collagen syntesis.^70^

Although our study did not uncover significant differences in epidermal thickness between the groups, it is noteworthy that a murine experimental model comparing polydioxanone, PCL, and PLA filler materials reported an increase in epidermal thickness.^71^ Similarly, another study utilizing HAc filler on human skin reported a 19% increase in epidermal thickness after 1 month.^72^ However, when the histomorphology of the epidermis, the outermost layer of the skin, is evaluated, it is unlikely that subcutaneous filler materials have a significant effect on epidermal thickening. Furthermore, studies assessing epidermal thickness did not explain this relatively small increase, which could be attributed to the stimulation of collagen synthesis of different types or an increase in the number of keratinocytes in the upper layer of the epidermis. Throughout the study, the tissue samples showed an increase in dermal thickness. The positive control and CaHA/CMC data exhibited similar characteristics at all periods. The literature associates increased dermal thickness with increased cell proliferation and collagen density, resulting in a denser and fuller dermal structure.^72^ Various studies attribute this biological response to HA particles across multiple filler materials, inducing fibroblast proliferation and activation.^69,73^ In addition, studies in the literature directly correlate the stimulation of new collagen synthesis in the dermis with dermal thickness, which plays an important role.^69,73,74^ In line with the literature, our current study revealed that the variable increase in dermal thickness was associated with increased fibroblast and collagen density during the same periods, supporting the pathophysiological explanation for dermal thickening.^65^ A study conducted by Marian et al. in aged rats reported that there were a limited number of weak elastic fibers in the dermis and almost none in the subcutaneous tissue. However, branched elastic fibers were prominently observed in the subcutaneous application area, where HAc was injected. In the same study, it was reported that on both day 7 and day 60, rats sacrificed after subcutaneous application of HAc showed the presence of long-branched parallel elastic fibers in the subcutaneous area.^63^ Conversely, our study found a statistically significant increase in the number of elastic fibers over time in the areas where our material was applied, in line with the results of our positive control group. In a similar study, new collagen and elastic fibers were observed to align at the tissue-filler interface in the injection areas, and the expression of dermal elastin protein was significantly increased in animals injected with HAc at 4, 8, and 12 weeks compared to the control group.^75^ In this context, our product, CaHA/CMC filler, is similar to the HAc effect emphasized in the literature. In a study using HAc-microHA and HAc-nanoHA as dermal filler materials, the density of elastic fibers was found to be higher than that in the Radiesse and Restylane groups used as controls.^67^ As the Radiesse filler material has the same content as CaHA/CMC in this study, it is conceivable that our material may be less effective in terms of elastic fiber density compared to materials containing HAc-microHA. However, a comparison in the literature suggests that HAc and HA have similar effects on fibroblast activation and elastic fiber synthesis. According to Fan et al., the combined use of HAc and microHA or nanoHA may enhance the success of the applied filler material in terms of elastic fiber synthesis compared to pure HAc and HA materials due to the synergistic effect of these two active materials.

The developed HA-based dermal filler boasts prolonged efficacy and biocompatibility, effectively stimulating collagen production for enduring improvements. Through enhancements in fluid properties, our product demonstrates superior longevity within the injected anatomical layer and enhanced resistance to environmental forces (Figure 3), contrasting some existing literature findings.^76,77^ Utilizing CMC as the carrier phase not only enhances mechanical stability due to the lack of cellulase enzyme in the human body but also leverages its plant-based origin to mitigate the risk of immune responses, setting it apart from animal-derived alternatives.^78,79^ Studies further affirm the effectiveness of HA-CMC fillers, particularly in hand augmentation, where they exhibit safety and efficacy akin to polymer-based fillers.^80,81^

## 5 Conclusions

In conclusion, our study presents a comprehensive evaluation of the novel CaHA/CMC gel formulation as a promising biomaterial for soft tissue repair and augmentation. Through rigorous characterization and *in vitro* and *in vivo* studies, we have demonstrated the efficacy, safety, and potential clinical utility of this innovative filler material. The CaHA/CMC formulation, integrating HA microspheres into CMC, exhibited favorable properties conducive to tissue integration and regeneration. Analytical techniques confirmed the purity of the HA component, while the amorphous nature of the carrier polymer phase suggested optimal rheological properties. The biocompatibility, nontoxicity, and biodegradability of the material, coupled with its ability to promote collagen and elastic fiber density, fibroblast activation, and dermal thickness, highlight its suitability for clinical use. Comparative analysis with literature findings underscored the effectiveness of HA-based fillers in stimulating tissue remodeling, with our formulation demonstrating comparable or superior outcomes. Importantly, the absence of adverse tissue reactions throughout the study period emphasizes the safety profile of the developed CaHA/CMC material, making it a reliable option for soft tissue augmentation procedures. Furthermore, the potential cost-effectiveness, ease of storage, and versatility in incorporating additional components, such as growth factors or drugs, enhance the clinical appeal of the CaHA/CMC gel filler material. Future research endeavors should focus on further validating its clinical efficacy, optimizing its formulation, and exploring innovative applications in tissue engineering and regenerative medicine.

In summary, the CaHA/CMC gel formulation represents a significant advancement in biomaterial science and is a versatile tool that can be recommended for clinical phase-by-phase studies to be evaluated in the addressing of soft tissue defects and achieving optimal aesthetic outcomes. With continued research and development, this innovative filler material holds great promise for revolutionizing the field of cosmetic and reconstructive surgery.

## Notes

**Ethical Approval** Experiments were performed according to the Guide for the Care and Use of Laboratory Animals published by the US National Institutes of Health (NIH publication No. 85–23, revised 1996). All experimental procedures in this study were approved by the Atatürk University Local Ethics Committee for Animal Experiments (No. 182, July 30, 2021).
